# Dysregulated FAM215A Stimulates LAMP2 Expression to Confer Drug-Resistant and Malignant in Human Liver Cancer

**DOI:** 10.3390/cells9040961

**Published:** 2020-04-14

**Authors:** Po-Shuan Huang, Yang-Hsiang Lin, Hsiang-Cheng Chi, Yi-Hsin Tseng, Cheng Yi Chen, Tzu-Kang Lin, Chau-Ting Yeh, Kwang-Huei Lin

**Affiliations:** 1 Department of Biochemistry, College of Medicine, Chang Gung University, Taoyuan 333, Taiwan, nonbalance@gmail.com (H.-C.C.); 2 Liver Research Center, Chang Gung Memorial Hospital, Linkou, Taoyuan 333, Taiwan, chauting@adm.cgmh.org.tw (C.-T.Y.); 3 Institute of Stem Cell and Translational Cancer Research, Chang Gung Memorial Hospital at Linkou, and Chang Gung University, Taoyuan 333, Taiwan; akiraest@yahoo.com.tw; 4 Department of Cell Biology and Anatomy, College of Medicine, National Cheng Kung University, Tainan 70101, Taiwan; cychen@gs.ncku.edu.tw; 5 Neurosurgery, Fu Jen Catholic University Hospital and School of Medicine, Fu Jen Catholic University, New Taipei City 24205, Taiwan; tklin100@cgmh.org.tw; 6 Research Center for Chinese Herbal Medicine, College of Human Ecology, Chang Gung University of Science and Technology, Taoyuan 333, Taiwan; 7 Graduate Institute of Biomedical Sciences, College of Medicine, Chang Gung University, Taoyuan 333, Taiwan

**Keywords:** HCC, Long non-coding RNA, FAM215A, Lysosome, LAMP2

## Abstract

Hepatocellular carcinoma (HCC) is one of the most common and aggressive human malignancies worldwide. Long non-coding (lnc) RNAs regulate complex cellular functions, such as cell growth, differentiation, metabolism, and metastasis. Although deregulation of lncRNA expression has been detected in HCC, many of the hepato-carcinogenesis-associated lncRNAs remain yet unidentified. Here, we aimed to investigate the involvement of a specific HCC-dysregulated lncRNA, FAM215A, and characterize its molecular regulation mechanism. We show for the first time that FAM215A is overexpressed in HCC, and its expression level correlates with tumor size, vascular invasion, and pathology stage. Overexpression of FAM215A accelerates cell proliferation and metastasis in HCC cells. According to Gene Expression Omnibus Dataset analysis, FAM215A is induced in doxorubicin (DOX)-resistant HCC cells. Overexpression of FAM215A increases DOX resistance in two HCC cell lines, and this is associated with enhanced expression of lysosome-associated membrane protein 2 (LAMP2). FAM215A interacts with LAMP2 to protect it from ubiquitination. Together, our results show that the lncRNA, FAM215A, is highly expressed in HCC, where it interacts with and stabilizes LAMP2 to increase tumor progression while decreasing doxorubicin sensitivity.

## 1. Introduction

Hepatocellular carcinoma (HCC) ranks among the most common cancers in many countries and is the second most common cause of cancer death worldwide. Most HCC is associated with liver cirrhosis that represents major risk factors (HBV- or HCV-related chronic liver disease, toxins and drugs) for HCC development, being implicated in more than 70% of HCC cases worldwide [1,2]. The lifestyle of a high-calorie, low-fiber diet and lack of exercise are highly correlated with obesity to cause non-alcoholic fatty liver disease (NAFLD). NAFLD covers a range of clinical diseases from hepatic steatosis to severe inflammatory steatohepatitis (NASH) [3]. A variety of genomic alterations have been identified in fully developed HCC. In the clinic, alpha-fetoprotein (AFP) is the main tumor biomarker available to guide the management of HCC; however, its low accuracy limits its usefulness as a screening test [4]. The survival rate of HCC is usually poor due to the high probability of tumor recurrence and metastasis after treatment. We therefore urgently need to identify novel therapeutic targets and biomarkers, with the goal of developing effective strategies to improve the prognosis of HCC [5].

Traditionally, studies of gene regulation have tended to focus on protein-coding genes. However, multiple lines of evidence from numerous high-throughput genomic platforms suggest that it more strongly reflects the regulatory potential of non-coding RNAs (ncRNAs) than the number of protein-coding genes, even when protein diversification by alternative splicing and post-translational regulation are taken into account [6,7].

Various small ncRNAs, such as siRNAs, miRNAs, and piRNAs, have been studied, and the research has shown that they are highly conserved and involved in transcriptional and posttranscriptional regulation of gene expression through specific base pairing with their targets. Another class of ncRNAs are the long non-coding RNAs (lncRNAs), which are operationally defined as RNAs larger than 200 base-pairs that do not “appear” to have coding potential, are poorly conserved, and regulate gene expression by diverse mechanisms that are complex and not yet fully understood [6].

Some lines of evidences indicate that most nuclear lncRNAs function by guiding chromatin modifiers to specific genomic locations. These lncRNAs recruit DNA methyltransferase 3 (DNMT3) and histone modifiers, such as the polycomb repressive complex (PRC2) and histone H3 lysine 9 (H3K9) methyltransferases. The resultant DNA and histone modifications predominantly correlate with repressive heterochromatin formation and transcriptional repression [7,8]. Many other lncRNA-mediated gene regulation mechanisms have been identified in the cytoplasm. The involved lncRNAs often show sequence complementarity with other transcripts; they may recognize the target by complementary base pairing, leading to modulation of translational processes. The target sites of lncRNAs differ between the so-called cis- and trans-acting lncRNAs; the former control the expression of genes that are positioned near the transcription site of that lncRNA, with their effects sometimes spreading across great distances on the same chromosome; in contrast, trans-acting lncRNAs can affect the expression of independently located genes [7,9].

Although few lncRNAs have been fully characterized, many have been shown to exhibit cell-specific expression and to be associated with cancer. Numerous studies have indicated that lncRNAs modulate cancer initiation and progression by affecting various biological pathways [10]. For example, lncRNAs have been reported to be aberrantly expressed in HCC and to play roles in modulating malignant phenotypes. Maternally expressed gene 3 (MEG3), which is an imprinted lncRNA located on chromosome 14q32.3 within the DLK-1 locus, is downregulated in 60% of HCC cases; this is correlated with increased DNA methylation, reduced tumor cell growth, and increased apoptosis [11]. HOX transcript antisense RNA (HOTAIR) is expressed from the developmental HOX-C locus located on chromosome 12q13.13, and was found to be up-regulated in patients with large primary HCC and those with nodal involvement. HOTAIR acts as an oncogene; it may positively regulate the expression levels of multiple genes involved in promoting metastatic processes, such as vascular endothelial growth factor (VEGF) and matrix metallopeptidase 9 (MMP9). HOTAIR acts as a scaffolding molecule that binds polycomb repressive complex 2 (PRC2) and lysine-specific demethylase 1 (LSD1) and increases the recruitment of enzymes involved in epigenetic modification to repress tumor suppressor genes [12,13,14].

Lysosomes are acidic (pH 4.5–5) catabolic organelles that are found in all mammalian cells. They are responsible for the disposal and recycling of worn-out and damaged cellular macromolecules and organelles, and the digestion of extracellular and foreign materials that are delivered to them by endocytosis, autophagy, and phagocytosis [15]. It has been well documented that during the process of cellular transformation and cancer progression, lysosomes exhibit changes in their localization, composition, and volume; moreover, lysozymes can, through the release of their enzymes, promote invasive growth, angiogenesis, and drug resistance [15,16]. Lysosomal-associated membrane protein (LAMP) 1 and 2 comprise nearly 50% of the lysosomal transmembrane, and they are essential for maintaining the structural integrity of the lysosomal compartment [15]. In addition to its expression on the lysosomal membrane, LAMP2 is reportedly relocalized to the cell surface of some highly metastatic tumor cells, such as melanoma and colon cancer cells [17,18]. In HCC, LAMP2 is required for tumor growth and promotes tumor recurrence [19]. Currently, we aimed to determine the function of a specific HCC-dysregulated lncRNA, FAM215A, and characterize its molecular action involvement of LAMP2 function.

## 2. Materials and Methods

### 2.1. Human HCC Specimens and Cancer Cell Lines

All clinical studies were performed in accordance with the approved guidelines of the Chang Gung Memorial Hospital Institutions Review Board (IRB: 201601542B0). Informed consent was obtained from all patients involved in this study. The human hepatoma cell lines, Hep3B, Mahlavu, and J7, were routinely grown in DMEM supplemented with 10% fetal bovine serum. Cells were cultured at 37 °C in a humidified atmosphere of 95% air and 5% CO_2_.

### 2.2. Quantitative Reverse Transcription-Polymerase Chain Reaction (qRT-PCR)

Total RNA was purified using the TRIzol reagent (Life Technologies, Carlsbad, CA, USA) according to the supplier’s protocol (MAN0016385), and cDNA was synthesized using a Superscript II kit (Life Technologies, Karlsruhe, Germany). The sequences of the primer pairs for FAM215A amplification were: forward primer, 5′-GCGGTAGCTCACCAATCCAA-3′, and reverse primer, 5′-CTCCTTATT TAACGCACTGTTGTATCA-3′. All reactions were conducted in an ABI PRISM 7500 sequencer (Applied Biosystems, Foster City, CA, USA). The other primers information for qRT-PCR were listed in the Supplementary Table S1.

### 2.3. Rapid Amplification of cDNA Ends (RACE)

A partial sequence of lncRNA-FAM215A was obtained from NCBI, and RACE PCR was performed to obtain the full-length sequence. Total RNA from Hep3B cells was used as the template for nested-PCR reactions using a 5’/3’-RACE Kit, 2nd Generation (Catalog No. 03353621001; Roche Applied Science, CA, USA, according to the manufacturer’s instructions. The products obtained from RACE PCR were cloned into a TA vector and sequenced.

### 2.4. Fractionation of Nuclear and Cytoplasmic RNA

To obtain nuclear and cytoplasmic RNA fractions, cells were washed twice with ice-cold PBS and centrifuged for 5 min at 290 g at 4 °C. Each cell pellet was resuspended in 1 mL RSB buffer (10 mM Tris-Cl [pH 7.4], 10 mM NaCl, and 3 mM MgCl2) and incubated for 3 min on ice. Cells were pelleted at 1500 g for 3 min at 4 °C, the supernatant was discarded, and the cells were lysed by gentle resuspension in 150 ul RSBG40 buffer (10 mM Tris-Cl [pH 7.4], 10 mM NaCl, 3 mM MgCl2, 10% glycerol, and 0.5% Nonidet P-40). Samples were centrifuged at 4500 g for 3 min at 4 °C. The supernatant (cytoplasmic fraction) was collected in a new Eppendorf tube, and 1 mL TRIzol reagent was added to each sample (nuclear pellet and cytoplasmic supernatant) to extract the relevant RNA fractions.

### 2.5. Establishment of FAM215A-Overexpressing Cells

The FAM215A plasmid was cloned into expression vector pLAS5w.PeGFP-I2-Bsd and prepared using an EasyPrep EndoFree Maxi Plasmid Extraction Kit (DPT-BA17; Biotools Co., Ltd., Taipei, Taiwan). The generated plasmid was transfected to Mahlavu and J7 cells using a lentivirus-based method and a FAM215A-encoding lentivirus (System Biosciences). The plasmid and lentiviral packaging plasmids were co-transfected to Hek-293T cells using a Turbofect Reagent Kit (Thermo Fisher Scientific, Waltham, MA, USA), and produced a virus in Hek-293T cell. The Mahlavu and J7 cells were infected with viruses collected from 24 h cultures of virus-producing Hek-293T cells. After 48 h of incubation, cells were transferred to medium containing blasticidin (3 μg/mL) for selection of infected cells, and FAM215A expression was confirmed by qRT-PCR.

### 2.6. Establishment of FAM215A-Knockdown Cells

The FAM215A shRNA plasmid was constructed by following the guidelines of the TRC shRNA Design Process from the National RNAi Core Facility (Institute of Molecular Biology, Academia Sinica, Taipei, Taiwan), and cloning the obtained shRNA sequence into the pLKO-TRC001 vector. The primers used to construct the sh-FAM215A#1 plasmid were: forward primer, 5′-CCGG GGTAGCTCACCAATCCAATGCCTCGAGGCATTGGATTGGTGAGCTACCTTTTT-3′; and reverse primer, 5′-AATTAAAAAGGTAGCTCACCAATCCAATGCCTCGAGGCATTGGA TTGGTGAGCT ACC-3′. Those used to construct the sh-FAM215A#2 plasmid were: forward primer, 5′-CCGGTTTGGGATGGTTGATTAGGACCTCGAGGTCCTAATCAACCATCCCAAATTTTT-3′; and reverse primer, 5′AATTAAAAATTTG GGATGGTTGATTAGGACCTCGAGGTCCTAATCAA CCATCCCAAA-3′. Each plasmid was transfected to Hep3B and J7 cells by a lentivirus-based method. The lentivirus of shRNA FAM215A was purchased from System Biosciences. The plasmid and lentiviral packaging plasmids were co-transfected to Hek-293T cells using the Turbofect Reagent, and produced virus in Hek-293T cell. After 24 h, viral soup was collected and used to infect Hep3B and J7 cells. After 48 h of incubation, the cells were transferred to a medium containing puromycin (0.3 μg/mL) for the selection of lentivirus-infected cells, and FAM215A expression was confirmed by qRT-PCR.

### 2.7. Establishment of LAMP2-Overexpressing and -Knockdown Cells

The LAMP2 plasmid was cloned into the pcDNA3 expression vector, which was transfected to FAM215A-knockdown Hep3B and J7 cells using the Turbofect Reagent. LAMP2 expression was confirmed by Western blot analysis. LAMP2 shRNAs were purchased from the National RNAi Core Facility (Institute of Molecular Biology, Academia Sinica, Taiwan). Stable LAMP2-knockdown Mahlavu and J7 cell lines infected with lentivirus-expressing shRNA against LAMP2 were established by culturing cells in selection medium consisting of DMEM with 10% fetal bovine serum and puromycin (0.3 μg/mL).

### 2.8. Wound-Healing Assay

To assess the cell migration capacity, 1.5 × 10^6^ cells/well were seeded to 6-well plates in 10% FBS DMEM and incubated for 24 h. A pipette tip was used to scratch (wound) the monolayer, and the medium was changed to 1% DMEM. The same positions were recorded under a microscope at 0 to 12 h post-wounding.

### 2.9. Invasion and Migration Assay

To assess cell invasive capacity, we used transwell chambers coated with (to assess invasion) or without (to assess migration) Matrigel (Collaborative Biomedical Products, Bedford, MA, USA). Briefly, 4 × 10^4^ cells in serum-free DMEM were seeded to the upper chambers of transwell filter devices; DMEM containing 20% FBS was added to the lower chambers, and the devices were incubated overnight. The next day, the migrated or invaded cells on the underside of the filter were stained with crystal violet and visualized with a microscope.

### 2.10. Cell Proliferation Assay

Cells were seeded at 3 × 10^4^ cells in a 6-well plate and incubated for 1 to 5 days. Staining with 0.4% Trypan Blue Solution was used to assess cell viability (viable cells excluded the dye), and a hemocytometer was used to count cell numbers.

### 2.11. Western Blot Analysis

Protein expression levels were determined by immunoblot analysis, which was performed according to a previous description [20]. Primary antibodies were obtained to detect LAMP2 (Abcam, Cambridge, MA, USA, ab-125068), TWIST (GeneTex, Irvine, CA, USA; GTX127310), SLUG, SNAIL, ERK, and phospho-ERK (Cell Signaling, Hitchin, UK; #9585, #3895, #4695 and #4370, respectively).

### 2.12. Ubiquitination assay

Cells were treated for 4 h with the proteasome inhibitor, MG132 (10 μM), and whole-cell lysates were extracted. The cell lysates were incubated with protein A (Santa Cruz Biotechnology, Santa Cruz, CA, USA) for 1 h to eliminate non-specific binding, and then with anti-LAMP2 (Abcam, Cambridge, MA, country, ab-125068) overnight at 4 °C. Total lysates were then incubated with protein A for 1 h at 4 °C and centrifuged, and the pelleted proteins were eluted from the beads by being heated in 50 µl of SDS loading buffer for 5 min. Ubiquitinated LAMP2 was detected by Western blot analysis using an anti-ubiquitin antibody (Santa Cruz Biotechnology; sc-8017).

### 2.13. Drug Resistance Assay

The MTT assay was used to assess the viability of cells treated with doxorubicin (5 μM), cisplatin (10 μM), or sorafenib (2 μM). The cells were seeded at 3 × 10^4^ cells in 96 well plate, grown for 1–5 days, and subjected to the MTT assay (Thiazolyl Blue Tetrazolium Bromide, 298-93-1, Sigma-Aldrich, St. Louis, MO, USA).).

### 2.14. Lysosomal Content Assay

Cells were incubated with LysoTracker Red dye (LysoTracker™ Red DND-99, L7528, Carlsbad, CA, USA) for 1 h at 37 °C in the dark, washed three times with PBS, and fixed in 3.7% formaldehyde for 10 min. Fluorescent images were captured and recorded using a fluorescent microscope and a CCD camera. The images were analyzed and quantified using the Image J software.

### 2.15. RNA Immunoprecipitation (RIP)

Cells were washed twice with ice-cold PBS, removed from the culture plate using a cell scraper, and placed in a 10-cm dish containing 1 mL of ice-cold polysomal lysis buffer (100 mM KCl, 5 mM MgCl2, 10 mM HEPES [pH 7.0], 0.5% NP40, 1 mM DTT, 50 U RNase inhibitor, and protease inhibitor). The resulting suspension was passed through a 27.5-G needle eight times to promote cell lysis. Whole-cell extracts were collected by centrifugation (12,000 rpm, 15 min) and pre-cleared with magnetic protein-A beads (Invitrogen) at 4 °C for 1 h. For immunoprecipitation, anti-LAMP2 was added to the pre-cleared extracts and the mixtures were incubated at 4 °C overnight. Magnetic protein-A beads were added to the IP samples and the mixtures were rotated for 1 h at 4 °C. The beads were pelleted and washed with polysomal lysis buffer. After several washes, 20 U of DNase I (Roche) and 10X reaction buffer was added and the mixtures were incubated at 37 °C for 15 min to remove contaminating DNA. Finally, 1 mL of TRIzol reagent was added to the beads and the RNA was extracted.

### 2.16. Mouse Xenograft Model

The metastatic ability of FAM215A-knockdown J7 cells was determined. Briefly, FAM215A-knockdown and shControl J7 cells (5 × 10^5^/100 μL PBS) were intravenously injected to the tail veins of severe combined immunodeficiency (SCID) mice (n = 5/group). All animals were sacrificed at 4 weeks after tumor inoculation, and lungs were removed. For establishment of solid tumors in nude mice (n = 5/group), 1 × 10^6^ stable FAM215A-knockdown J7 cells/100 μL PBS were subcutaneously injected and tumor growth was measured. When J7 shControl tumors reached >50 mm3 in volume, mice were injected intraperitoneally with doxorubicin (4 mg/kg body weight) twice per week. Animal experiments were performed in accordance with the United States National Institutes of Health guidelines and the Chang-Gung Institutional Animal Care (Taoyuan, Taiwan) and Use Committee Guide for Care and Use of Laboratory Animals.

### 2.17. Statistical Analysis

Statistical analyses were carried out using means and standard deviations, one-way analysis of variance (ANOVA), and Tukey’s honest significant difference post-hoc test.

## 3. Results

### 3.1. lncRNA FAM215A Is Up-Regulated in Hepatocellular Carcinoma and Is Associated with Tumor Progression and Metastasis

**Figure 1:**
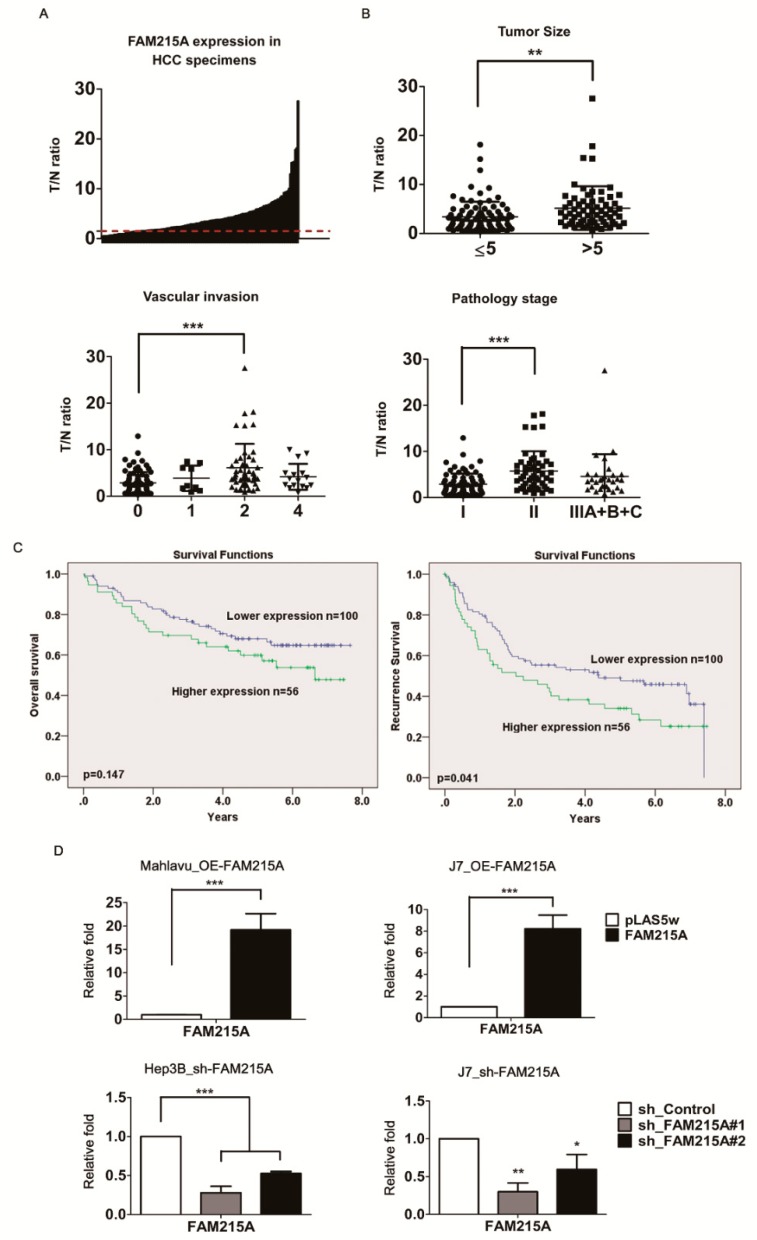
FAM215A dysregulated in HCC and correlated with HCC tumorigenesis. To verify the role and expression of FAM215A in 156 pairs of HCC clinical specimens by qRT-PCR. (**A**) FAM215A is highly expressed in 156 pairs of HCC specimens. (**B**) Clinical significance of FAM215A. (**C**) Overall survival or recurrence free survival. The average value of expression is the cutoff (high or low). (**D**) Overexpression or depletion of FAM215A cells by using lentivirus. Expression of FAM215A was determined by qRT-PCR. 18s rRNA was used as the loading controls. Data are presented as means ± s.d. (*p < 0.05; **p < 0.01; ***p < 0.001) of three independent experiments. Data show as tumor/adjacent normal (T/N ratio). Vascular invasion: 0. Absent, 1. Capsular vein invasion, 2. Portal vein tumor thrombosis (micro), 3. Portal vein tumor thrombosis (grossly) and 4. Portal vein tumor thrombosis (gross and micro). Pathology stage is according to TNM stage: Stage I. T1, Stage II. T2 and Stage III. T3-4. The square, triangle and circle are used to indicate the labels of different pathological groups.

Our previous microarray analysis identified the lncRNA, FAM215A, as being highly expressed and dysregulated in human hepatoma samples [21]. To verify the alteration of FAM215A expression in HCC, 156 paired HCC tissues and corresponding adjacent normal tissues were collected and qRT-PCR was used to assess the levels of FAM215A. Indeed, FAM215A expression was found to be significantly upregulated in 78% (123 of 156, ≧ 1.5 fold) of the cancerous tissue samples compared with their normal counterparts (Figure 1A). The sequence of FAM215A is presented in Figure 1B. The encoding gene is located on chromosome 17 in humans; it consists of a single 773-nt exon, as determined by RACE (rapid amplification of cDNA ends) assay in Hep3B cells (Supplemental Figure S1A). To examine the physiological significance of FAM215A overexpression in HCC, we assessed whether it was associated with various clinicopathological features of HCC patients. Indeed, high FAM215A expression was significantly correlated with tumor size, vascular invasion, and pathology stage in HCC, and FAM215A upregulation was also closely correlated with the recurrence-free survival of HCC patients (Figure 1B,C). The genes encoding lncRNAs constitute a large fraction of the mammalian transcriptome, yet relatively few lncRNAs have been subjected to a comprehensive, high-quality annotation of their gene structure and boundaries [22]. Similar to proteins, the functions of lncRNAs can depend on their subcellular localization [23]. Here, we found that FAM215A is expressed in both nuclear and cytoplasmic fractions of Mahlavu and J7 cells. In these experiments (Supplemental Figure S1B), GAPDH was used as a cytoplasmic marker and U1 was used as a nuclear marker.

### 3.2. FAM215A Promotes HCC Cell Metastasis and Proliferation

**Figure 2:**
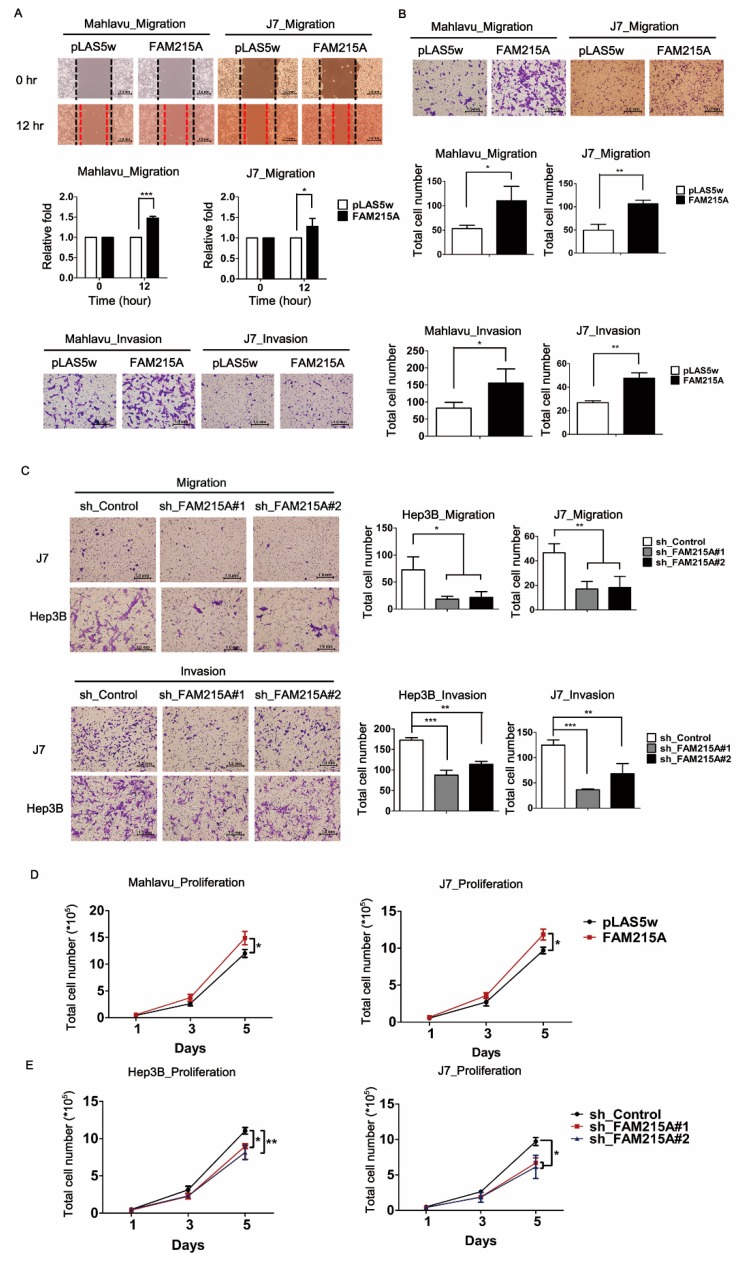
FAM215A promotes cell metastasis and proliferation in HCC. Migration and invasion capacity in FAM215A-expressing or depletion cells were determined by (**A**) Wound healing assay, (**B**) Transwell assay in Mahlavu and J7 cell lines. (**C**) Migration and invasion ability assayed by transwell in Hep3B and J7 cell lines. (**D**,**E**) Proliferation rate measured by the total cell numbers (1-5days). Data are presented as means ± SD of three independent experiments (*, *p* < 0.05 ; **, *p* < 0.01 ; ***, *p* < 0.001).

To investigate the potential role of FAM215A in HCC cells, we established stable expression lines using Mahlavu and J7 cells. qRT-PCR assays verified that FAM215A expression was significantly increased in the stable expression lines (Figure 1D). A wound-healing assay showed that overexpression of FAM215A significantly increased cell migration in Mahlavu and J7 cells compared with the corresponding control cells (Figure 2A). Similarly, a transwell assay revealed that migration and invasion were significantly increased in Mahlavu and J7 cells overexpressing FAM215A (Figure 2B), as were cell metastasis and cell proliferation (Figure 2D). We generated Hep3B and J7 cells with stable knockdown of FAM215A, as verified by qRT-PCR assays (Figure 1E), and found that the significant depletion of FAM215A decreased cell metastasis and proliferation (Figure 2C, E). We also assessed the epithelial-mesenchymal transition (EMT), which is classically associated with the relocation of cells from a basement membrane microenvironment into a fibrillar ECM [24,25]. Upon knockdown of FAM215A, many EMT-related transcription factors, such as SNAIL, SLUG, and TWIST, were repressed, as assessed by Western blot analysis (Supplemental Figure S1C). Extracellular signal-related kinase 1/2 (ERK1/2) is a member of the mitogen-activated protein kinase (MAPK) family and is reportedly associated with cell proliferation [26]. Interestingly, knockdown of FAM215A repressed the phosphorylation of ERK (Supplemental Figure S1E). Our findings clearly indicate that FAM215A plays an oncogenic role in HCC cell lines.

### 3.3. FAM215A Promotes Doxorubicin Resistance and Is Highly Expressed in Doxorubicin-Resistant HCC Cells

**Figure 3:**
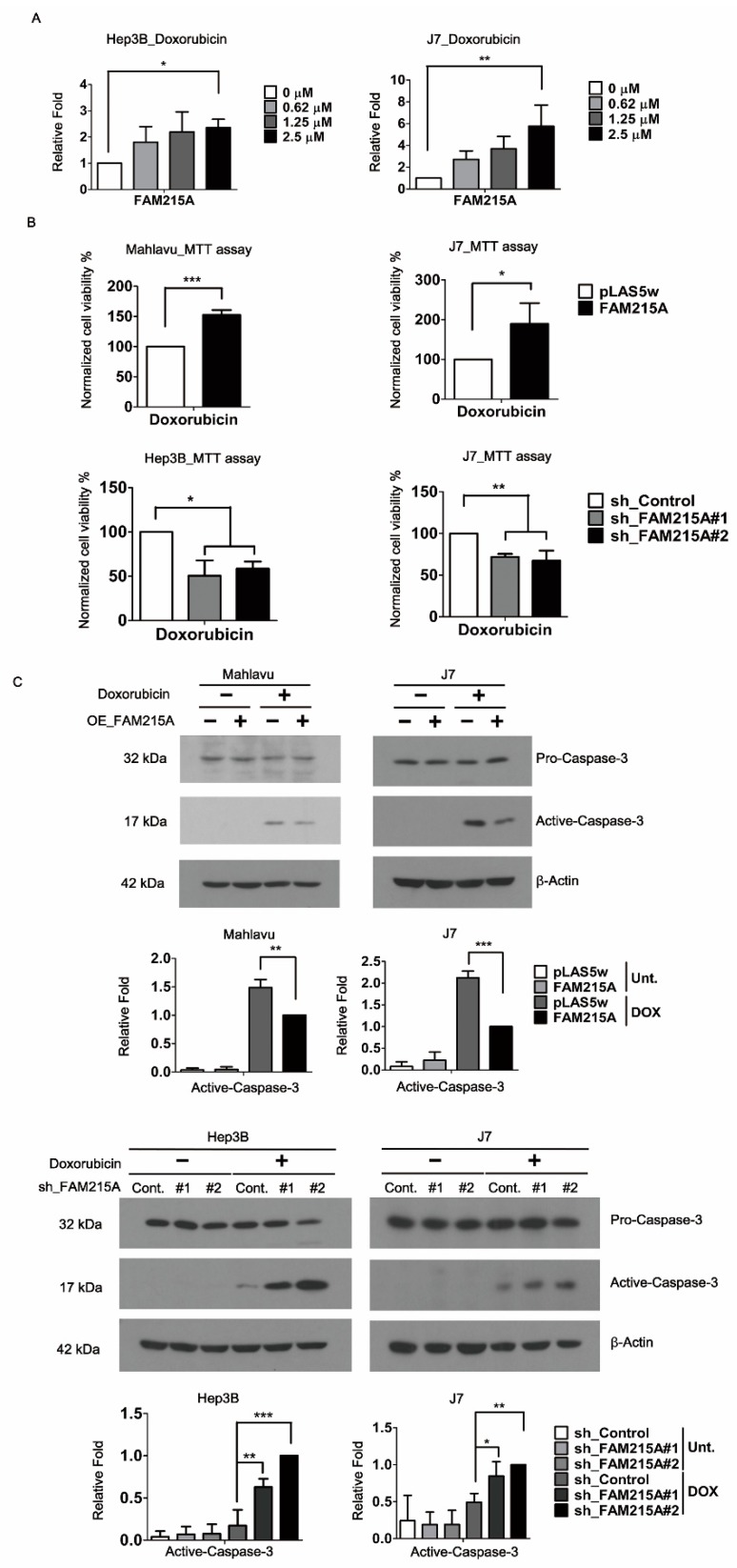
FAM215A is regulated by Doxorubicin and inhibits apoptosis. (**A**) Doxroubicin (0–2.5 μM) regulates FAM215A expression in Hep3B and J7 cells determined by qRT-PCR. (**B**) Cell viability treated with Doxorubicin with/without FAM215A expression. The data are normalized to the untreated groups. (**C**) Expression of apoptosis marker active caspase-3 and the pro-caspase-3 detected in FAM215A-expressing or knockdown HCC cells treated with Doxroubicin 5 μM for 24 h by western blot. 18s rRNA and β-Actin was used as the loading controls. Data are presented as means ± SD of three independent experiments (*, *p* < 0.05; **, *p* < 0.01; ***, *p* < 0.001).

Chemoresistance is a major obstacle limiting the success of systemic chemotherapy and targeted therapy for patients with advanced HCC. Doxorubicin (DOX) is one of the most widely used anti-HCC drugs for chemotherapy [27]. Analysis of the Gene Expression Omnibus (GEO) datasets [28] revealed that FAM215A is specifically induced in DOX-resistant HCC cells (7.24-fold) but not in cisplatin (CP)-resistant HCC cells. To verify this result, we used qRT-PCR to examine the expression of FAM215A in Hep3B and J7 cells treated with various doses of DOX. Our results revealed that doxorubicin treatment induced FAM215A by ~1.8–2.2-fold and 2.3–5.5-fold in Hep3B and J7 cells, respectively (Figure 3A). To ascertain the importance of FAM215A to drug resistance in HCC cells, we performed MTT assays on FAM215A-overexpressing and -knockdown HCC cells treated with Doxorubicin. Indeed, our results revealed that FAM215A increases the Doxorubicin resistance of HCC cells (Figure 3B). As Doxorubicin induces apoptosis by activating caspase-3 [29], we assessed the level of activated caspase-3 in Doxorubicin-treated FAM215A-overexpressing and -knockdown HCC cells. Our results indicated that FAM215A represses the Doxorubicin-induced activation of caspase-3 in the tested HCC cell lines (Figure 3C). Our findings indicate that FAM215A promotes Doxorubicin resistance in HCC cells.

### 3.4. FAM215A Increases HCC Progression and Drug Resistance through LAMP2

**Figure 4:**
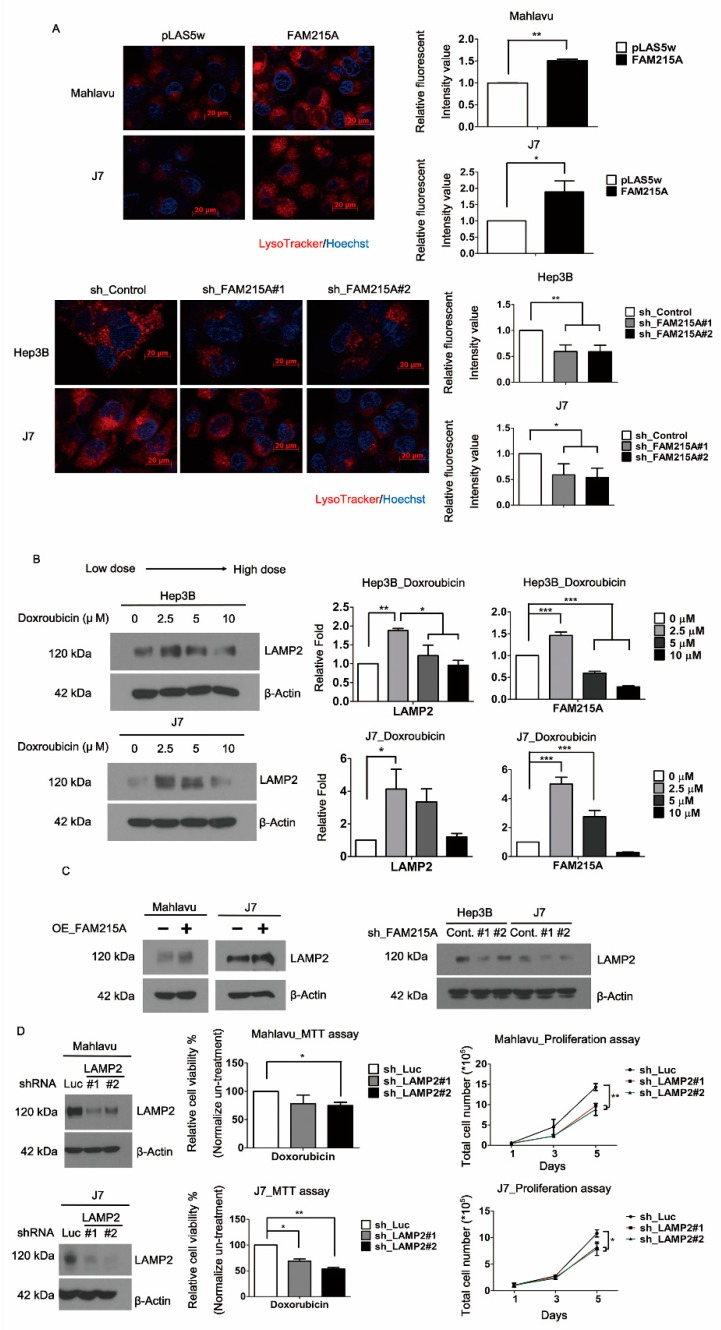
FAM215A affects lysosomes and the oncogene LAMP2 expression in HCC. (**A**) Lysosome expression (red fluorescent) in FAM215A-expressing or knockdown J7 cells determined by LysoTracker. Blue fluorescent is Hoechst that indicates nucleus. The signal was detected by fluorescence microscopy images and analysis with ImageJ. (**B**) The effects of Doxroubicin (0–10μM, 24hours) on the expression of FAM215A and LAMP2 detected by Western blot and qRT-PCR. (**C**) Expression of LAMP2 in FAM215A-expressing or knockdown cells assayed by Western blot. (**D**) Proliferation rate measured by MTT in LAMP2-depleted Mahlavu and J7 cells with/without 5 μM Doxroubicin for 24 h. All the data are normalized with un-treatment groups. Data are presented as means ± SD of three independent experiments (*, *p* < 0.05 ; **, *p* < 0.01 ; ***, *p* < 0.001).

**Figure 5:**
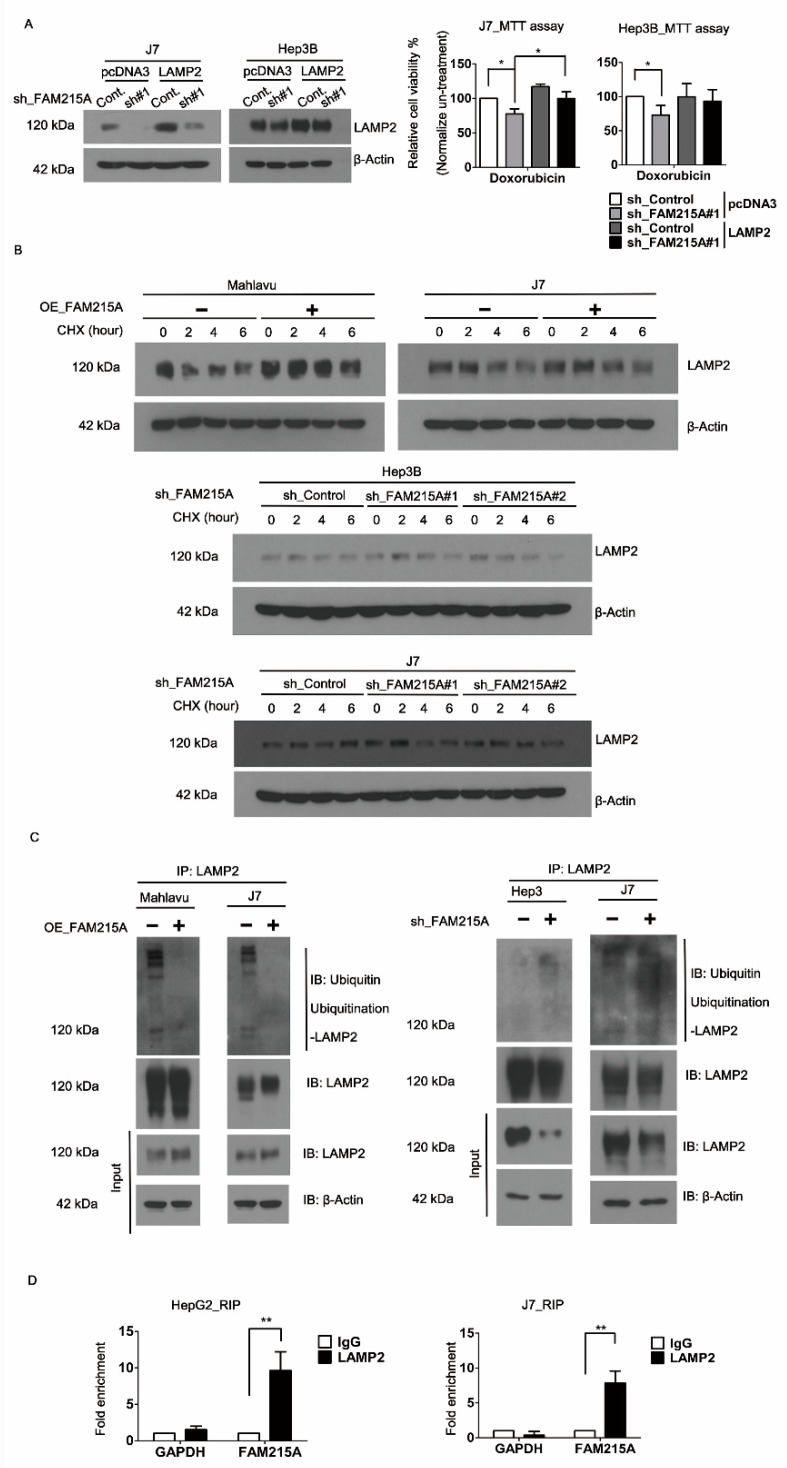
FAM215A confers Doxroubicin resistance in HCC by affecting the LAMP2 stability. (**A**) To restore LAMP2 expression in FAM215A knockdown of Hep3B and J7 cell lines detected by Western blot. The cell viability assay was performed on MTT assay with Doxroubicin 5μM 24 h. (**B**) The protein stability of LAMP2 was assessed by treatment CHX 10 μg for 1–6 h in FAM215A-expressing or knockdown cells detected by Western blot. (**C**) The ubiquitination of LAMP2 was assessed by immunoprecipitation (IP) that was treated with MG132 for 4 h in FAM215A-expressing or knockdown cells. (**D**) Interaction of FAM215A and LAMP2 assessed by RNA immunoprecipitation (RIP) in HepG2 and J7 cells, IgG as the control antibody and GAPDH as the negative control. β-Actin and 18s rRNA were used as the loading controls. Data are presented as means ± SD of three independent experiments (*, *p* < 0.05; **, *p* < 0.01).

Similarly, cells treated with Cisplatin or Sorafenib in FAM215A-overexpression or -knockdown conditions, the same results that are consistent with the treated doxorubicin can be obtained by MTT assay (Supplemental Figure S1D, E). However, cisplatin did not affect FAM215A expression (Supplemental Figure S1F). Based on the FAM215A influence on different drugs’ sensitivity, we examined the lysosome expression of strong correlation with the multidrug resistance (MDR) of cancer cells [30]. We found that overexpression and knockdown of FAM215A will positively affect the lysosomal content, as assessed by LysoTracker (Figure 4A). Lysosomes are membrane-bound intracellular organelles that receive macromolecules delivered by endocytosis, phagocytosis, and autophagy for degradation and recycling [31]. The lysosomal membranes contain several highly N-glycosylated proteins, including LAMP1 and LAMP2 [32]. LAMP2A expression in HCC contributes to tumor cell viability and promotes tumor growth and recurrence. Thus, it has been speculated that LAMP2A could be targeted as a novel treatment strategy for HCC [19]. Indeed, inhibition or genetic knockdown of LAMP2A resulted in the sensitization of tumor cells to Doxorubicin and radiation therapy [16]. Here, we found that FAM215A was affected by different doses of Doxorubicin (Figure 3A). We also observed that LAMP2 and FAM215A exhibited a positive correlation in Hep3B and J7 cells treated with different doses of Doxorubicin (Figure 4B). The protein expression level of LAMP2 was increased in FAM215A-overexpressing HCC cells and decreased in FAM215A-knockdown HCC cells (Figure 4C). To further verify the role of LAMP2 in HCC cells, we established LAMP2-depleted Mahlavu and J7 cells. The proliferation and viability of LAMP2-knockdown cells were reduced by Doxorubicin treatment (Figure 4D). To address the possible involvement of LAMP2 in the FAM215A-mediated promotion of doxorubicin resistance, LAMP2 was re-expressed in FAM215A-knockdown J7 and Hep3B cells. Whereas the knockdown of FAM215A in J7 and Hep3B cells inhibited their Doxorubicin resistance, this parameter was restored following the re-expression of LAMP2 (Figure 5A). Our findings indicate that FAM215A increases LAMP2 expression to induce Doxorubicin resistance in HCC cells.

### 3.5. FAM215A Interacts with and Stabilizes LAMP2

To further investigate the mechanism by which FAM215A affects LAMP2 expression, we used cycloheximide (CHX) treatment to examine the protein stability of LAMP2 in FAM215A-knockdown and -overexpressing cells. We found that the protein stability of LAMP2 was repressed by FAM215A knockdown and increased by FAM215A overexpression in J7 or Mahlavu cells (Figure 5B). To test whether this stability change involved proteasome-mediated degradation, we assessed the ubiquitination of LAMP2 in MG132-treated FAM215A-overexpressing or -knockdown HCC cells. We found that LAMP2 ubiquitination was decreased in FAM215A-overexpressing J7 or Mahlavu cells, whereas it was increased in FAM215A-repressing Hep3B and J7 cells (Figure 5C). We further used an RNA immunoprecipitation (RIP) assay to determine whether FAM215A and LAMP2 interacted in HepG2 or J7 cells, and found that FAM215A interacts with LAMP2 (Figure 5D) to increase its protein stability in HCC cells.

### 3.6. FAM215A Increases HCC Progression and Drug Resistance In Vivo

**Figure 6:**
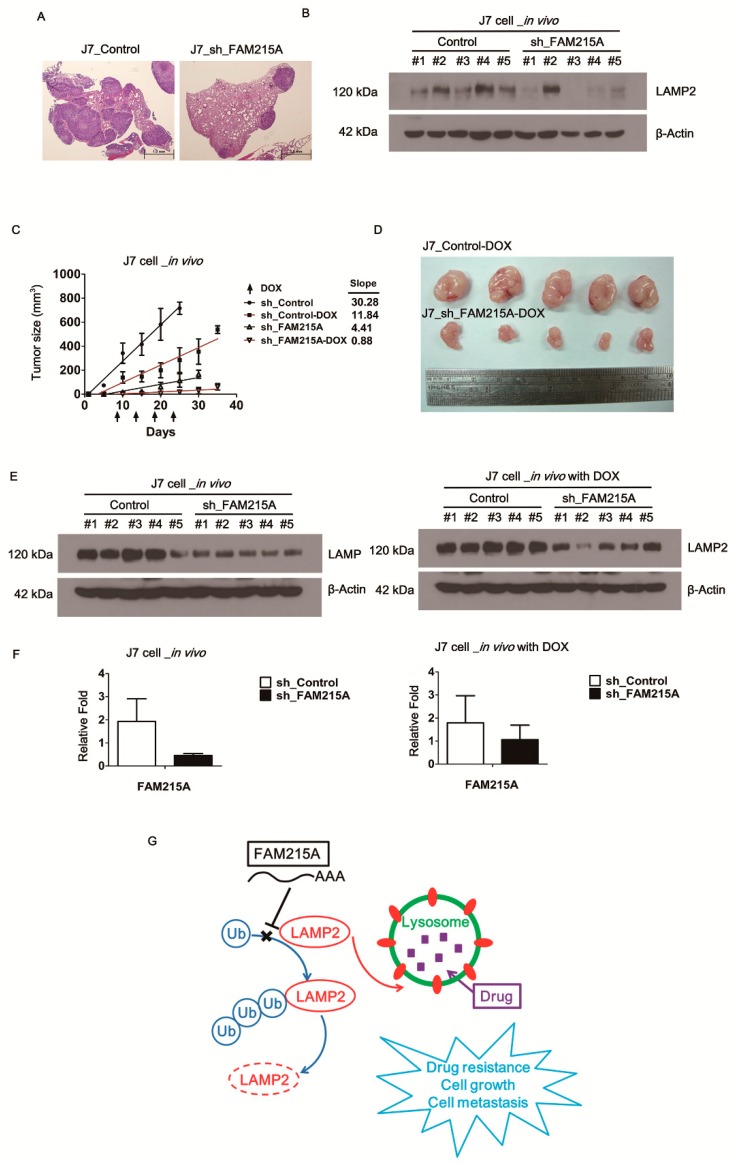
FAM215A promotes tumor progression and drug resistance in vivo by xenograft mouse. (**A**) Injection of the FAM215A knockdown in J7 cells by tail vein in severe combined immunodeficiency (SCID) mice (n = 5). The hematoxylin and eosin (H&E) staining of the tumor in lung tissues was used to determine the metastasis capacity. (**B**) LAMP2 expression from the tumor in the lung by Western blot. (**C**,**D**) Affect FAM215A on the tumor growth curve (slope) and drug resistance capacity treated with Doxroubicin (4 mg/kg). FAM215A depleted in J7 cells were subcutaneously injected into nude mice (n = 5). (**E, F**) Expression of the LAMP2 and FAM215A determined by Western blot or qRT-PCR. β-Actin and 18s rRNA were used as the loading controls. Arrow indicates the time of Doxroubicin treatment. Data are presented as means ± SD of five independent experiments. (**G**) Diagram of FAM215A inhibits LAMP2-ubiquitination to confer drug-resistant in HCC.

To determine whether the in vitro effects of FAM215A could be replicated in vivo, we used xenograft mouse models. Indeed, tumors derived from FAM215A-knockdown J7 cells administered by tail vein injection exhibited less tumor metastasis compared to those of mice injected with control cells (Figure 6A). In metastatic tumors found in the lungs of such mice, LAMP2 expression was decreased in mice with FAM215A-knockdown cell-derived tumors compared to mice injected with control cells (Figure 6B). To validate the role of FAM215A in tumor formation and DOX resistance, FAM215A-depleted J7 cells were subcutaneously injected into nude mice. Our results revealed that mice injected with FAM215A-knockdown cells displayed decreased tumor growth compared to mice injected with control cells (Figure 6C). We also examined whether stable knockdown of FAM215A increased the DOX sensitivity of J7 cell-derived tumors in mice. DOX was administered twice a week via intraperitoneal injection, beginning when the tumor volume of the J7 control group reached ~50 mm^3^. Indeed, our data showed that FAM215A knockdown was associated with decreased tumor growth and further repression of the tumor size in DOX-treated mice. The slope of the growth curve was decreased nearly 51% (slope, Control-DOX 11.84/30.28 versus sh-FAM215A-DOX 0.88/4.41) (Figure 6C,D). The expression levels of FAM215A and LAMP2 were decreased in the tumor tissues with FAM215A-knockdown cell-derived tumors compared to mice injected with control cells (Figure 6E,F). Together, our findings clearly indicate that FAM215A increases tumor progression and DOX resistance in HCC by interacting with and increasing the stability of LAMP2 (Figure 6G).

## 4. Discussion

Recent studies have shown that lncRNAs have important functions in modulating various tumor progression in HCC, such as growth arrest-specific 5 (GAS5), nuclear-enriched autosomal transcript 1(NEAT1), and taurine upregulated gene 1 (TUG1) [21,33,34]. FAM215A is a novel dysregulated lncRNA that our previous microarray analysis identified as being highly expressed in human hepatoma tissues [21]. Conversely, in ovarian cancer, FAM215A is associated with favorable overall survival [35]. In the current study, we show that FAM215A is highly expressed in human hepatoma samples and associated with clinical markers of tumor progression, including tumor size, vascular invasion, and pathologic stage. Moreover, high-level expression of FAM215A in HCC is associated with a poor recurrence-survival ratio. We obtained consistent results when we analyzed data from the Cancer Genome Atlas (TCGA) using the cBio Cancer Genomics Portal [36]. Further, FAM215A leads to HCC cell metastasis, and up-regulation of EMT-related transcription factors, including TWIST, SNAIL, and SLUG, is seen in FAM215A-overexpressing J7 and Hep3B cells. The latter finding is relevant because EMT-related phenotypes have been found to exhibit significant associations with clinicopathological factors indicating aggressive biologic behavior and poor outcome in patients with liver cancer and other types of cancer [37,38,39]. In addition to cell metastasis, FAM215A increases cell growth and induces the phosphorylation of ERK1/2, in which it has been shown that growth factor could enhance cell proliferation and survival through the activation of the ERK1/2 pathway [40]. ERK1/2 also plays an important role in the metabolism of the liver. ERK1/2 can affect liver metabolism by positively affecting the activation of Peroxisome proliferator-activated receptors (PPARs)-alpha, a gene related to liver fat metabolism [41,42]. PPAR is a ligand-activated transcription factor that regulates mitochondrial function, ATP generation, antioxidant, immune responses, and fatty acid oxidation [43].

Doxorubicin is one of the most widely used drugs for chemotherapy in HCC [44]. Doxorubicin mainly plays a role in chemotherapy cancer through two different mechanisms of action. (I) Doxorubicin intercalates into DNA and interference of topoisomerase II-mediated DNA repair. (II) Doxorubicin generation of reactive oxygen species (ROS) leads to lipid peroxidation and membrane damage, DNA damage, oxidative stress, and severe inflammatory responses to triggers apoptotic pathways of cell death [45,46]. In the analyzed Gene Expression Omnibus (GEO) datasets, FAM215A is a highly expressing gene in doxorubicin-resistant HCC cells [28]. Here, we show that FAM215A is specifically regulated by doxorubicin, but not cisplatin. This suggests that FAM215A may act as a survival factor under Doxorubicin therapy in HCC. Several other genes have been found to be upregulated in drug-adapted cells. For example, NGFR is highly expressed in cells exposed to vemurafenib (a RAF inhibitor), allowing these cells to transiently exit the cell cycle and induce an adaptive response, thereby making them drug-resistant. Interestingly, pathway enrichment analysis showed that various pathways found to be altered by upregulation of NGFR (e.g., the cell adhesion, ECM remodeling, and EMT pathways) are also altered by the upregulation of other factors, including the ECM component, thrombospondin-1 (TSP-1), adhesive glycoprotein, laminin subunits LAMA1 and LAMC1, and several integrin family receptors. Additionally, integrin β1 is regulated in a manner similar to FAM215A when cells are treated with different dose drugs [47]. The lncRNA promoter of CDKN1A antisense DNA-damage-activated RNA (PANDAR) has been reported to serve as a negative regulator of cisplatin sensitivity, and cisplatin has been shown to induce higher levels of PANDAR than doxorubicin or paclitaxel in human ovarian cancer cells [41]. Moreover, PANDAR exhibited higher expression in cisplatin-resistant ovarian cancer tissues and cells, compared with cisplatin-sensitive tissues and cells, and mechanistic studies showed that it negatively regulates cisplatin sensitivity in human ovarian cancer via PANDAR-SRFS2-p53 feedback regulation in the nucleus [48]. By analogy, these studies strengthen our concept regarding the relationship between FAM215A and doxorubicin in liver cancer cells. Here, we clearly show that FAM215A increases the resistance of HCC cells to doxorubicin by mechanisms that go beyond simply increasing cell proliferation and the capacity for metastasis.

In addition to its regulation of doxorubicin sensitivity, FAM215A also affects the sensitivity of cancer cells to other anticancer drugs, such as cisplatin and sorafenib. We thus analyzed a mechanism of action that is known to be related to multiple drug resistance (MDR). Lysosomal sequestration (or lysosomal drug entrapment) of anti-cancer compounds reduces drug availability at intracellular target sites, thereby limiting drug sensitivity and inducing chemoresistance [30,49]. Lysosomes mediate MDR in many cancers, such as those showing resistance to cisplatin, sorafenib, doxorubicin, and oxaliplatin [30]. Lysosomes recycle organelles and proteins in cells [50] and they play extensive roles in various diseases, including cancers, making them an attractive and targetable node for therapeutic intervention. In liver cancer, lysosomes are known to be involved in the chemoresistance of HCC cells [51,52]. Here, we used LysoTracker probes to assess the lysosomal contents of cells and found that FAM215A had a positive effect on the lysosomal content of HCC cells. Mechanistically, the lysosomal membrane proteins, LAMP1 and LAMP2, are estimated to contribute to about 50% of all lysosome membrane proteins [53]. LAMP2 has been shown to play roles in many diseases, but few such studies have been performed in liver cancer [16,54,55]. Here, we report that doxorubicin had similar effects on the expression levels of FAM215A and LAMP2. Indeed, we generally observed a positive correlation between FAM215A and LAMP2 in HCC cells. In previous studies, LAMP2 was shown to act as an oncogene, to be required for tumor growth, and to promote tumor recurrence [19]. Consistent with our data, LAMP2 knockdown in HCC cells repressed doxorubicin resistance. Our results therefore collectively suggest that FAM215A induces tumor progression, metastasis, and doxorubicin resistance, potentially through LAMP2. Consistent with this proposal, re-expression of LAMP2 in FAM215A-depleted HCC cells rescued their doxorubicin resistance.

To explore a mechanism through which FAM215A could affect LAMP2 protein expression, we used the eukaryotic translation inhibitor, cycloheximide (CHX), to assesses changes in the protein stability of LAMP2 in our various cell lines. Our data showed that FAM215A increased LAMP2 protein stability. This result was further confirmed by assessing the ubiquitination of LAMP2 in MG132-treated cells. Our findings indicated that FAM215A prevents the ubiquitination of LAMP2 to decrease its degradation. Moreover, RNA immunoprecipitation (RIP) showed that FAM215A and LAMP2 interact. Similarly, previous work showed that the lncRNA, metastasis-associated lung adenocarcinoma transcript 1 (MALAT1), interacts with SREBP-1c to stabilize nuclear SREBP-1c protein and thereby promote hepatic steatosis and insulin resistance [56]. We propose that FAM215A acts analogously to stabilize LAMP2 and alter downstream functions. The glycoprotein-specific F-box protein, FBXO27, is part of the SCF (SKP1/CUL1/F-box protein) ubiquitin ligase complex, and is also important for the ubiquitination of LAMP2. The N-myristoylation of FBXO27 localizes it to membranes, allowing it to accumulate rapidly around damaged lysosomes. Meanwhile, LAMP2 is ubiquitinated and degraded to enhance recruitment of the autophagic machinery to damaged lysosomes [57]. Finally, we used animal models to confirm that the in vitro effects of FAM215A can be replicated in vivo. Our results collectively indicate that FAM215A induces tumor metastasis, enhances tumor growth, and increases the doxorubicin resistance capacity in HCC.

Currently, we report that FAM215A increases the ability of liver cancer cells to resist different anti-cancer drugs. At the same time, FAM215A improves the ability of LAMP2, which plays an important role in the process of autophagy, and also seems to imply a new treatment strategy to overcome the failure of anti-cancer drug treatment caused by autophagy [30,58,59]. In conclusion, we herein show for the first time that the lncRNA, FAM215A, is highly expressed in HCC, where it interacts with and prevents the ubiquitination of LAMP2 to increase tumor progression and decrease doxorubicin sensitivity.

## Supplementary Materials

The following are available online at https://www.mdpi.com/2073-4409/9/4/961/s1, Figure S1: FAM215A information and its role with cisplatin and sorafenib in HCC., Table S1: Primer sets used for qRT-PCR analysis.
