# Supplementary material for: Dysregulated FAM215A Stimulates LAMP2 Expression to Confer Drug-Resistant and Malignant in Human Liver Cancer
Source: Cells. 2020 Apr 14;9(4):961. doi: 10.3390/cells9040961 (PMC7227021; doi:10.3390/cells9040961)
Supplement: Supplementary file 1 [file cells-09-00961-s001.zip › cells-776176-supplementary/Supplementary Table S1.docx]

Table S1. Primer sets used for qRT-PCR analysis.

| Gene | Forward Primer (5' to 3') | Meting temperature (℃) | Reverse Primer (5' to 3') | Meting T (℃) | Product legth | Gene bank code |
| --- | --- | --- | --- | --- | --- | --- |
| 18s rRNA | CGAGCCGCCTGGATACC | 62.8 | CCTCAGTTCCGAAAAC  CAACAA | 63.4 | 76 bp | XR_004381726.1 |
| FAM215A | GCGGTAGCTCACCAATC  CAA | 63.4 | CTCCTTATTTAACGCAC  TGTTGTATCA | 61.8 | 101 bp | NR_026770.1 |
| LAMP2 | AAACCAGAAGCTGGAA  CCTATTCA | 62.4 | GAAGCAACCTTATCCTG  AGTGATGT | 62 | 101 bp | NM_002294.3 |
