# Supplementary figures and images for: Dysregulated FAM215A Stimulates LAMP2 Expression to Confer Drug-Resistant and Malignant in Human Liver Cancer
Source: Cells. 2020 Apr 14;9(4):961. doi: 10.3390/cells9040961 (PMC7227021; doi:10.3390/cells9040961)

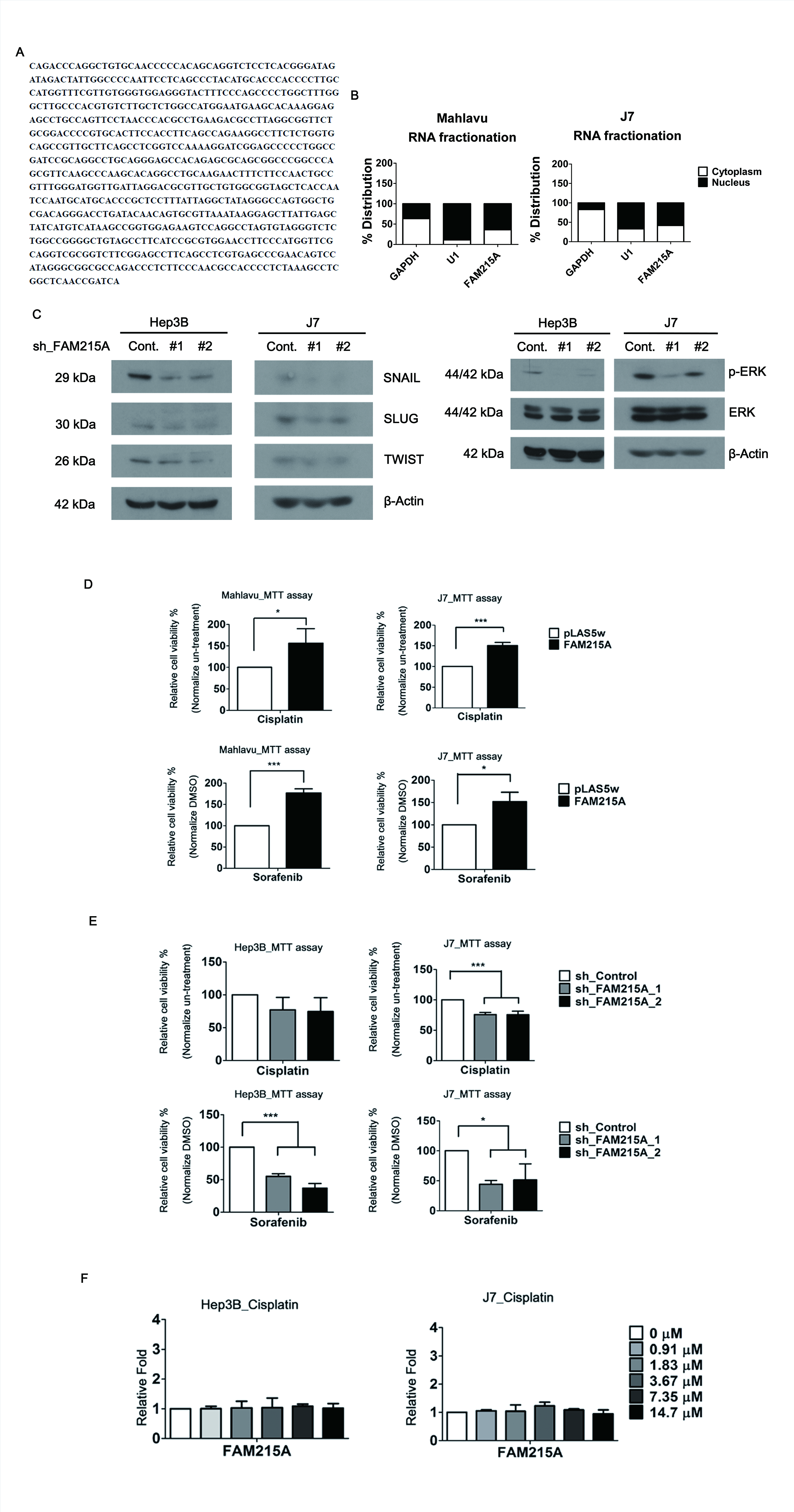

Supplement: Supplementary file 1 [file cells-09-00961-s001.zip › cells-776176-supplementary/Supplementary Figure S1 .tif]
